# Excitation–contraction coupling of human induced pluripotent stem cell-derived cardiomyocytes

**DOI:** 10.3389/fcell.2015.00059

**Published:** 2015-09-29

**Authors:** Christopher Kane, Liam Couch, Cesare M. N. Terracciano

**Affiliations:** Laboratory of Cell Electrophysiology, National Heart and Lung Institute, Imperial College LondonLondon, UK

**Keywords:** induced pluripotent stem cell-derived cardiomyocytes, excitation–contraction coupling, stem cell maturation, disease modeling, pharmacological screening

## Abstract

Induced pluripotent stem cell-derived cardiomyocytes (iPSC-CMs) hold enormous potential in many fields of cardiovascular research. Overcoming many of the limitations of their embryonic counterparts, the application of iPSC-CMs ranges from facilitating investigation of familial cardiac disease and pharmacological toxicity screening to personalized medicine and autologous cardiac cell therapies. The main factor preventing the full realization of this potential is the limited maturity of iPSC-CMs, which display a number of substantial differences in comparison to adult cardiomyocytes. Excitation–contraction (EC) coupling, a fundamental property of cardiomyocytes, is often described in iPSC-CMs as being more analogous to neonatal than adult cardiomyocytes. With Ca^2+^ handling linked, directly or indirectly, to almost all other properties of cardiomyocytes, a solid understanding of this process will be crucial to fully realizing the potential of this technology. Here, we discuss the implications of differences in EC coupling when considering the potential applications of human iPSC-CMs in a number of areas as well as detailing the current understanding of this fundamental process in these cells.

## Introduction

Induced pluripotent stem cells (iPSCs) were first described by Shinya Yamanaka and colleagues in 2006 who detailed the reprograming of adult mouse fibroblasts into a pluripotent state (Takahashi and Yamanaka, 2006). The means to generate *de novo* cardiomyocytes from iPSCs was then described in mice in 2008 (Mauritz et al., 2008; Narazaki et al., 2008) and later in human (Zhang et al., 2009). Since then, interest in induced pluripotent stem cell-derived cardiomyocytes (iPSC-CMs) has steadily increased. iPSC-CMs represented a step-change in stem cell research, surmounting ethical issues surrounding consent and the use of embryonic material that has restricted research using embryonic stem cells (ESCs), making them a much more attractive technology to expand into wider research and industry. Furthermore, as iPSCs and their derivatives are specific to the genetic makeup of the cell donor, whole avenues of research open up in terms of personalized medicine and familial disease, applications that could not have been considered with human ESC-CMs (hESC-CM).

Excitation–contraction (EC) coupling is not only one of the fundamental properties of cardiomyocytes, turning rhythmical electrical stimulation into the production of mechanical force, but is also central to determining many other electrophysiological and mechanical cell properties and is a key process that becomes dysregulated in almost all cardiac diseases. Studies of EC coupling in human iPSC-CMs (hiPSC-CMs) have described substantial differences between their properties and those of adult cardiomyocytes. While understanding these differences will be crucial to fully realizing the potential of hiPSC-CMs, research to date is limited. Furthermore, electrophysiological study of hiPSC-CMs is confounded by the variability of these cells in culture. We have previously shown the markedly increased variability in action potential morphology when hiPSC-CMs are cultured as single cells as compared to in a monolayer. As patch clamping studies necessitate the use of individual cells, these findings raise questions over the usefulness of investigations performed in this manner (Du et al., 2015).

In this mini-review we discuss the importance of EC coupling when considering the full potential of hiPSC-CMs in a wide range of fields, as well as detailing our current understanding of the components of this fundamental property of cardiomyocytes.

## Excitation–contraction coupling in

### Drug discovery and screening

The development of new pharmacological agents is a long and hugely expensive process, taking up to 10 years to bring a novel agent to market with an average cost of $2.6 billion in 2014 (Mullard, 2014). In spite of the massive investment involved in this process, FDA approvals of “new chemical entities” are steadily declining (Paul et al., 2010). Up to 90% of compounds that pass pre-clinical screening fail at the highly expensive clinical trial level, with around one third showing unforeseen side-effects (Kola and Landis, 2004). Cardiotoxicity alone accounts for 45% of post-approval withdrawal of compounds (Stevens and Baker, 2009). Improvements to pre-clinical screening of novel compounds for efficacy and toxicity would provide an enormous boon to the pharmaceutical industry, both in terms of reducing expensive attrition rates and improving safety.

The main weakness of pre-clinical screening is the use of *in vitro* and animal models that do not sufficiently reproduce human physiology and disease. As such hiPSC-CMs are a highly attractive option when considering replacements for current models. There has been much discussion about the suitability of hiPSC-CMs in this application (Khan et al., 2013; Sharma et al., 2013; Sinnecker et al., 2014), however there is still concern that hiPSC-CMs do not recapitulate adult human physiology enough to be useful (Jonsson et al., 2012). Furthermore, while industry utilizes a wide range of assays to assess drug effects, many rely directly or indirectly on the underlying process of EC coupling. An understanding of iPSC-CM Ca^2+^ handling is therefore crucial to properly interpret these experiments and consider their validity in the context of adult pharmacological therapy.

### Cell therapy

A further exciting application of hiPSC-CMs is in the realm of cell therapy, potentially providing an autologous cell source that could be used in the treatment of a wide range of pathologies. As has been discussed elsewhere (Chen et al., 2009), there are a number of substantial electrophysiological challenges that need to be overcome to successfully employ hiPSC-CMs in cell therapy. The arrhythmogenic risks of using electrophysiologically immature cardiomyocytes in cell therapy has been demonstrated in detail (Zhang, 2002; Chang et al., 2006; Lalit et al., 2014; Smit and Coronel, 2014). As such a firm understanding of the EC coupling properties of these cells will be important in overcoming the risks and improving the benefits of cell therapy techniques utilizing hiPSC-CMs.

### Disease modeling

A third major area that has benefited from iPSC technology is that of disease modeling and in particular the modeling of inherited cardiac diseases. Since their discovery, iPSC-CM lines for a wide range of pathologies have been developed, such as channelopathies (Liu and Trudeau, 2015; Ma et al., 2015); cardiomyopathies (Caspi et al., 2013; Lin et al., 2015); as well as mutations in Ca^2+^ handling machinery such as ryanodine receptors and calsequestrin (Novak et al., 2012; Di Pasquale et al., 2013). Still, there is concern over the ability of these cells to truly recapitulate the diseases they model (Bellin et al., 2012; Blazeski et al., 2012; Rajamohan et al., 2013). Indeed investigators modeling arrhythmogenic right ventricular dysplasia using hiPSC-CMs described how the induction of more mature metabolic properties was essential to accurately model adult-onset cardiac disease (Wen et al., 2015). Therefore, understanding the current electrophysiological properties of hiPSC-CMs will be crucial for proper interpretation of investigations into diseases modeled in this way.

## Calcium handling in induced pluripotent stem cell-derived cardiomyocytes

**Figure 1:**
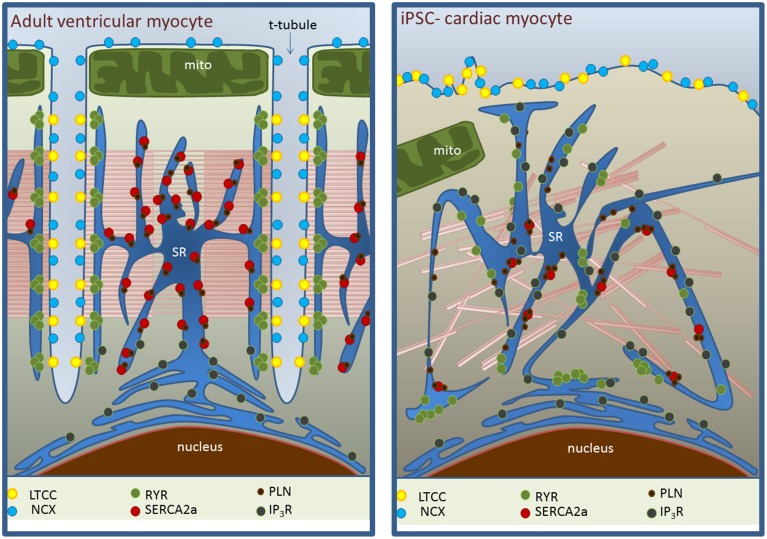
**Ultrastructural differences between adult myocytes (left) and iPSC-CMs (right) in EC coupling domains**. The absence of t-tubules in iPSC-CM is associated with the lack of regular organization of L-type Ca^2+^ channel (LTCC)-RyR complexes and less homogeneous distribution of RyRs. SERCA expression is reduced with a maintained phospholamban (PLN) expression. NCX expression may be maintained but its ability to extrude Ca^2+^ in diastole is decreased (see main text). Inositol-triphosphate receptor (IP_3_R) activity is substantially higher.

Ca^2+^-induced Ca^2+^ release (CICR) is the key link between electrophysiological stimulation and the production of mechanical force as well as a feature that distinguishes cardiomyocytes from other types of muscle cells. Cardiomyocyte depolarization causes Ca^2+^ entry via voltage-sensitive L-type Ca^2+^ channels, triggering the release of Ca^2+^ from the sarcoplasmic reticulum (SR) via ryanodine receptors (RyR), raising [Ca^2+^]_*i*_ and causing contraction. Relaxation is achieved by removing the Ca^2+^ from the cytosol, either by re-sequestering it in the SR via the sarco-endoplasmic reticulum ATP-ase (SERCA), or extruding it into the extracellular space through the Na^+^-Ca^2+^ exchanger (NCX) or Ca^2+^ ATPase pump (Bers, 2002; Figure 1).

While hiPSC-CMs show robust Ca^2+^ transients, they rise and decline at a substantially slower rate (Lee et al., 2011; Hwang et al., 2015). The rate of decline of Ca^2+^ transients assessed in a number of iPSC-CM lines showed κ-values of 0.84–1.15 s^−1^ compared to 2.41 and 5.75 s^−1^ in adult rabbit and mouse cardiomyocytes respectively (Hwang et al., 2015). Exhibiting a strong adrenergic response (Yokoo et al., 2009; Germanguz et al., 2011), hiPSC-CMs display a negative force-frequency response (FFR; Germanguz et al., 2011), suggesting reduced basal SERCA function and an inability to utilize more of their Ca^2+^ stores.

## L-type Ca^2+^ channels

L-type Ca^2+^ channels are a vital component of EC coupling in hiPSC-CMs. We have previously shown that CaV_1.2_, the α-1C subunit of L-type Ca^2+^ channels, demonstrates similar expression levels to the adult human heart (Rao et al., 2013). Application of the Ca^2+^ channel blocker nifedipine and removal of [Ca^2+^]_*o*_ both completely suppress whole cell Ca^2+^ transients in these cells, demonstrating that external Ca^2+^ is crucial for triggering SR Ca^2+^ release (Itzhaki et al., 2011). A number of studies investigating Ca^2+^ current (I_*Ca*_) through patch clamp techniques have measured substantial peak current densities, ranging from −8 pA/pF (Zhang et al., 2013b) to −17.1±1.7 pA/pF at RT (Ma et al., 2011), large in comparison to H1 hESC-CMs (Sartiani et al., 2007), although similar to mouse cardiac myocytes (Hwang et al., 2015).

hiPSC-CMs respond to a wide range of Ca^2+^ channel antagonists in a similar manner to both H7 hESC-CMs and guinea pig myocytes (Kang et al., 2012). However, hiPSC-CMs display unusual responses to Ca^2+^ channel agonists. Bay K8644 has little effect on current amplitude (Kang et al., 2012; Ji et al., 2014), while responses to the Ca^2+^ channel activator FPL 64176 produced an increase in current in some cells but a reduction in others (Kang et al., 2012). Hyperphosphorylation of channels in heart failure is known to reduce the effect of Bay K8644 (Chen et al., 2008) and may explain these unusual effects in hiPSC-CMs. Understanding differences in channel pharmacology is crucial for the use of hiPSC-CMs in drug screening platforms.

## The sarcoplasmic reticulum

### Ryanodine receptors

High conductance Ca^2+^ sensitive channels in the SR membrane, ryanodine receptors (RyRs) are the Ca^2+^ release mechanism of the SR, and are closely apposed to L-type Ca^2+^ channels. RYR2, the cardiac isoform of RyRs, appear diffuse throughout the cytosol on immunostaining with reports of both intense, punctate perinuclear (Itzhaki et al., 2011) and sub-sarcolemmal (Zhang et al., 2013b) staining. Expression is significantly lower in hiPSC-CMs than in H7 and HES3 hESC-CMs (Lee et al., 2011) and the adult human heart as well as levels of its regulatory proteins calsequestrin and triadin (Rao et al., 2013).

RyRs in hiPSC-CMs exhibit rapid, large releases of Ca^2+^ in response to the agonist caffeine (Fatima et al., 2011; Itzhaki et al., 2011; Lee et al., 2011; Kim et al., 2015; Zhang et al., 2015), and antagonism with ryanodine reduces whole cell Ca^2+^ transient amplitude. SR Ca^2+^ content, assessed by caffeine application, is similar to that found in adult rabbit cells (Hwang et al., 2015).

Activation of RyRs by Ca^2+^ entering the cell through L-type Ca^2+^ channels is crucial to the generation of mature calcium transients and there is evidence for functional CICR in hiPSC-CMs. hiPSC-CM transient amplitude reduced in a dose dependent manner in response to treatment with the selective L-type Ca^2+^ channel blocker nifedipine, abolishing transients altogether at 1 μM (Itzhaki et al., 2011). Further, Ca^2+^ transients in these cells appear to be voltage dependent, with transient amplitude correlating positively with the size of the depolarization step used to activate I_*Ca*_. Transients were elicited regardless of the direction of voltage change, consistent with I_*Ca*_ activation on depolarization and re-activation of I_*Ca*_ channels on repolarization. This dependence on I_*Ca*_ rather than the direction of voltage change distinguishes cardiac CICR from voltage-induced Ca^2+^ release particular to skeletal muscle (Zhang et al., 2013b).

The initial rise in [Ca^2+^]_*i*_ in I_*Ca*_-induced Ca^2+^ release has been observed to occur at the sarcolemma before spreading inwards, suggesting inefficient CICR, with more internal, non-coupled RyRs being activated by the subsequent rise in [Ca^2+^]_*i*_ as opposed to direct activation by Ca^2+^ channels (Lee et al., 2011; Zhang et al., 2013b). While a detailed EM study of iPSC-CM ultrastructure identified some apposition of the SR with the sarcolemma (Gherghiceanu et al., 2011), hiPSC-CMs exhibit poor co-localization between CaV_1.2_ and RYR2 (Rao et al., 2013).

Ca^2+^ sparks are small releases of Ca^2+^ from the SR generated by the stochastic opening of small clusters of RyRs and represent the fundamental release unit of the CICR. In keeping with the spatial variability noted with global Ca^2+^ transients, Ca^2+^ sparks were observed to occur repeatedly at the same sites within the cell, with their properties differing between those occurring at the center vs. the periphery of the cell (Zhang et al., 2013a), suggesting an inhomogeneous clustering of RyR channels, as seen on immunofluoresence. The majority of sparks demonstrated multiple peaks in three-dimensional and temporal profiles, suggesting that more than one RyR cluster may be involved in each release event. Spark characteristics were comparable with those of adult rat cardiomyocytes.

### SERCA and phospholamban

SERCA2a, the cardiac isoform of SERCA, is expressed in hiPSC-CMs at significantly lower levels compared to both hESC-derived myocytes (H7 and HES3 lines; Itzhaki et al., 2011; Lee et al., 2011) and the adult human heart (Rao et al., 2013).

Functionally, the rate of SR Ca^2+^ uptake is substantially slower in hiPSC-CMs than in adult rabbit and mouse cardiomyocytes (0.49–0.72 vs. 1.52 and 5.12 s^−1^ at RT; Hwang et al., 2015). Phospholamban is a key regulator of SR function, inhibiting SERCA activity when in its unphosphorylated form. Interestingly PLN expression is comparable to adult human cardiomyocytes (Rao et al., 2013); a high PLN:SERCA ratio may explain the low basal activity (Germanguz et al., 2011), and the concurrent ability of hiPSC-CMs to mount a strong response to adrenergic stimulation (Yokoo et al., 2009; Germanguz et al., 2011).

### Inositol-triphosphate receptors

IP_3_R antagonists 2-APB and U73122 produced a significant reduction in transient amplitude in hiPSC-CMs (Itzhaki et al., 2011). Prominent IP_3_ activity is a common feature of neonatal (Poindexter et al., 2001) and failing adult human cardiomyocytes (Go et al., 1995; Ai et al., 2005). However, maximal application of U73122 completely inhibited Ca^2+^ transients, somewhat in conflict with the identical observation in the same study with high concentration thapsigargin, an inhibitor of SERCA. It has been suggested that U73122 has off target effects on SR Ca^2+^ uptake (Macmillan and McCarron, 2010) but there is also evidence to support a complex role for IP_3_-mediated Ca^2+^ release in stem cell-derived Ca^2+^ handling. In mouse CMV ESC-CMs, inhibition of IP_3_ production with U73122 was associated with a decrease in spontaneous activity and Ca^2+^ spark frequency, while stimulation with ET-1 had positive chronotropic effects and increased diastolic [Ca^2+^]_*i*_ in the presence of tetracaine (Kapur and Banach, 2007). Whilst it is unclear exactly how IP_3_-mediated Ca^2+^ release works together with other Ca^2+^ handling pathways, future work will likely further highlight the importance of this mechanism in iPSC-CM Ca^2+^ cycling.

## The sodium–calcium exchanger NCX

Expressed as the NCX1 isoform in the heart, NCX has a key role in maintaining Ca^2+^ homeostasis, moving Ca^2+^ out across the sarcolemma during systole in exchange for Na^+^ moving into the cell at a ratio of 3:1 (3 Na^+^ ions in for each Ca^2+^ ion out). Due to this stoichiometry, NCX generates a substantial inward current (I_*NCX*_) that contributes to cell depolarization, pace maker activity and action potential duration (Blaustein and Lederer, 1999). Linking Ca^2+^ cycling and cell membrane potential, NCX is one key factor in afterdepolarization-based arrhythmogenesis and is as such of great interest as both a therapeutic target and a source of pharmacological toxicity (Pott et al., 2011).

Expression of NCX in hiPSC-CMs is reduced compared to two ESC-derived lines of cardiomyocytes (H7 and HES3; Lee et al., 2011), although more recently we have shown expression comparable to that in the adult human heart (Rao et al., 2013). NCX currents generated on application of 2 mM Ca^2+^showed peak densities of 4.5 ± 0.5 pA/pF at 37°C compared to an average of < 0.5 pA/pF in neonatal rat cardiomyocytes (Fine et al., 2013). A similar effect was seen in peak I_*NCX*_ elicited on application of 10 mM caffeine, with peak densities of ~2.6 pA/pF in hiPSC-CMs at 24°C vs. ~0.8 pA/pF in neonatal rat cardiomyocytes (Zhang et al., 2015), correlating with reports in additional investigations of ~2.2 pA/pF in hiPSC-CMs (Zhang et al., 2013b). KB-R7943 reduced caffeine-induced current amplitude (Zhang et al., 2015), demonstrating the ability of the exchanger's forward mode to influence resting membrane potential. To date there has been no assessment of [Na^+^]_*i*_ or the reversal potential of the exchanger in hiPSC-CMs and as such the possibility of substantial Ca^2+^ influx via reverse mode NCX remains unexplored.

Regarding the relative contribution of the two main mechanisms, SR Ca^2+^ uptake and NCX, to Ca^2+^ extrusion, Hwang et al. have recently reported that this is similar to adult rabbit myocytes, with approximately 60% of the Ca^2+^ released by caffeine removed by the SR and 40% by NCX, which may suggest robust and adequate diastolic function (Hwang et al., 2015). However, as previously discussed, the rate of decline of Ca^2+^ transients is extremely slow in hiPSC-CMs with both absolute values for SR Ca^2+^uptake and NCX- mediated Ca^2+^ extrusion significantly reduced. This also stands in contrast to the consistent observation that hiPSC-CMs display a negative FFR, a phenomenon observed in the failing heart, consistent with substantially reduced SR function. The similarity in relative contribution to Ca^2+^ extrusion may be peculiar to the experimental conditions used (room temperature, 0.5 Hz stimulation rate) and as such need to be interpreted with caution. In summary, hiPSC-CMs display significantly impaired diastolic Ca^2+^ removal.

### Spontaneous SR Ca^2+^ release events and NCX

A key feature of hiPSC-CMs is that they beat spontaneously, making for a convenient means to confirm successful differentiation of stem cells into cardiomyocytes. Unexpectedly, study of this phenomenon has given us a number of insights into the regulation of Ca^2+^ handling in these cells.

Optical techniques assessing spontaneous SR Ca^2+^ release identified small Ca^2+^ wavelets propagating within the cell, the majority of which were too small to trigger a global Ca^2+^ release (Kim et al., 2015). Successive wavelets appeared to originate repeatedly from the same sites throughout the cytosol, an observation also made above when looking at Ca^2+^ sparks, which increased in amplitude to a point where a global Ca^2+^ release was triggered.

Beating rate of the cells correlated positively with the application of isoproterenol and negatively with reduced [Ca^2+^]_*o*_, as did the incidence of Ca^2+^ wavelets (Kim et al., 2015). The RyR stabilizer K201 (Kim et al., 2015), inhibitors tetracaine and ryanodine (Kim et al., 2015; Zhang et al., 2015), the NCX inhibitor SEA0400 (Kim et al., 2015) and SERCA2a inhibitor thapsigargin (Zhang et al., 2015) all reduced or abolished spontaneous activity in hiPSC-CMs, confirming that automaticity in these cells was dependent on spontaneous SR release generating a depolarizing current via NCX. Further, a combination of switch-clamp recording of membrane potential and GCamP6-FKBP, a Ca^2+^-sensing probe targeted at RyRs, showed that Ca^2+^ release events correlated with diastolic depolarization preceding the action potential upstroke (Zhang et al., 2015).

## Mitochondrial Ca^2+^

The topic of mitochondrial Ca^2+^ in cardiomyocytes and its consequences for Ca^2+^ cycling is subject to intense debate. While it is well accepted that oscillations in mitochondrial Ca^2+^ occur during cytosolic Ca^2+^ transients (Boyman et al., 2014), there is conflicting evidence as to whether mitochondria play an active or passive role in calcium cycling, be that through changes in ATP production (Luongo et al., 2015), Ca^2+^ release (Zhao et al., 2013) or buffering of cytosolic Ca^2+^ transients (Drago et al., 2012; Boyman et al., 2014). A number of excellent reviews have been written on the subject (Lukyanenko et al., 2009; Kohlhaas and Maack, 2013).

In hiPSC-CMs, exposure to the mitochondrial uncoupler FCCP, at low concentrations reported to enhance mitochondrial oxidative respiration without changing membrane potential (Brennan et al., 2006), abolished the occurrence of spontaneous Ca^2+^ transients (Zhang et al., 2015). This stands in stark contrast to the finding in mouse ventricular cardiomyocytes that identical concentrations of FCCP enhance spontaneous SR release by releasing Ca^2+^ from mitochondria (Zhao et al., 2013). The ability of mitochondria to affect SR release is suggested to be due to the formation of functional micro-domains between the two structures, where small movements of Ca^2+^ can produce large changes in local concentration (Lu et al., 2013). The limited ultrastructural organization of hiPSC-CMs may mean that these micro-domains are lost.

However, in the same study, the genetically encoded mitochondrial Ca^2+^ sensing probe mitycam-E31Q revealed that, during spontaneous depolarization, mitochondrial Ca^2+^ dynamics displayed regional variability, with the perinuclear mitochondrial population releasing Ca^2+^ while peripheral organelles sequestered Ca^2+^ (Zhang et al., 2015). While the authors do not provide evidence as to whether this mitochondrial Ca^2+^ movement precedes or is a consequence of SR Ca^2+^ release, the correlation between mitochondrial Ca^2+^ release, cellular micro-domains prone to spontaneous Ca^2+^ release events and the perinuclear generation of whole cell Ca^2+^ transients suggests mitochondrial activity plays a role in regulating Ca^2+^ cycling in these cells.

## Structure-function relationships in hiPSC-CMs

The lack of the sophisticated adult myocyte structure in hiPSC-CMs may explain some functional observations. From studies discussed here it is clear that SR Ca^2+^ release is limited in Ca^2+^-induced transients, with fractional release, calculated as ΔF_(*ICa*)_/ΔF_(*Caff*)_, averaging at ~0.3 vs. 0.6–0.9 in adult human cells (Bassani et al., 1995; Zhang et al., 2013b). Inhomogeneous clustering of RyRs, the consequent loss of local control of Ca^2+^ release and limited association with sarcolemmal Ca^2+^ channels contributes to slow and inefficient rise of [Ca^2+^]_*i*_. hiPSC-CMs are devoid of t-tubules (Gherghiceanu et al., 2011), the sites of dyad formation in the cardiomyocyte. The absence of t-tubules in adult cells, in atrial or failing cardiomyocytes for example, correlates with a number of properties we have discussed here in hiPSC-CMs such as sub-sarcolemmal generation of Ca^2+^ transients, low fractional release and increased dependence on sarcolemmal Ca^2+^ flux (Figure 1). This structural immaturity may be a limiting step in these cells becoming more functionally mature.

Equally so the opposite can be argued. T-tubule formation and maintenance is an inherently energy-intensive process. Low SERCA expression and Ca^2+^ extrusion rate, high PLN:SERCA ratio and a negative force-frequency response point to decreased SR function and regulation, associated with a greater dependence on sarcolemmal flux to source and extrude Ca^2+^. As such, greater structural co-ordination may yield limited benefits without improvements in the underlying function of EC coupling components, and vice-versa.

Standard culture environments place little demand on hiPSC-CMs in terms of Ca^2+^ handling, and as such it could be argued that it is more efficient to rely on sarcolemmal flux driven by electrochemical gradients than ATP-intensive processes. We and others have shown how using culture techniques to manipulate myocyte structure can result in substantial changes in Ca^2+^ handling properties (Kaji et al., 2003; Yin, 2004; Rao et al., 2013; Trantidou et al., 2014).

## Conclusion and future directions

hiPSC-CMs hold enormous promise in many fields of cardiovascular research. While we have a reasonable understanding of the Ca^2+^ handling properties of these cells a number of questions remain, particularly with regards to how well Ca^2+^ channels and RyRs are coupled and what the dominant sources of Ca^2+^ are in EC coupling.

With many of the end applications of hiPSC-CMs dependent on the ability of these cells to recapitulate adult human cardiomyocyte properties to a satisfactory degree, differences in channel pharmacology, mechanisms of automaticity and structural organization between hiPSC-CMs and adult cardiomyocytes pose challenges to fully realizing the potential of this technology. It is important however to remember that there is no truly definitive understanding of human cardiac physiology. Difficulties in obtaining healthy adult tissue have meant that the field has come to rely on failing hearts, and in particular on rabbit physiology, as a basis of comparison. While this by no means invalidates the comparative studies above nor the concerns raised about hiPSC-CM function, it is essential to consider this limitation when discussing hiPSC-CM properties.

From a more practical perspective, the minimal variability shown between different cells lines and different differentiation protocols is reassuring, and gives us greater confidence when comparing and collating the work of multiple groups. Consideration must be given however to culture conditions, with substantial variability in hiPSC-CM electrophysiology apparently dependent on cell density (Du et al., 2015). Further work will be needed to determine the general applicability of studies that necessitate the use of single cells and to develop more suitable alternatives.

Overall, a more solid understanding of the electrophysiological properties and limits of hiPSC-CMs will be extremely valuable in the interpretation of the effects of novel drugs and making judgments on the ability of these cells to successfully recapitulate human disease.

## Authorship

We confirm that CK, LC, and CT meet the criteria for authorship of this review based on the recommendations of the ICMJE.

## Funding

CK is funded by a British Heart Foundation MBBS/PhD studentship FS/13/46/30282.

### Conflict of interest statement

The authors declare that the research was conducted in the absence of any commercial or financial relationships that could be construed as a potential conflict of interest.
